# Fetal Heart Sounds Detection Using Wavelet Transform and Fractal Dimension

**DOI:** 10.3389/fbioe.2017.00049

**Published:** 2017-09-08

**Authors:** Elisavet Koutsiana, Leontios J. Hadjileontiadis, Ioanna Chouvarda, Ahsan H. Khandoker

**Affiliations:** ^1^Laboratory of Medical Informatics, The Medical School, Aristotle University of Thessaloniki, Thessaloniki, Greece; ^2^Department of Electrical and Computer Engineering, Aristotle University of Thessaloniki, Thessaloniki, Greece; ^3^Department of Electrical and Computer Engineering, Khalifa University of Science and Technology, Abu Dhabi, United Arab Emirates; ^4^Department of Biomedical Engineering, Khalifa University of Science and Technology, Abu Dhabi, United Arab Emirates

**Keywords:** fetal heart rate, fetal heart sound, fetal phonocardiogram, wavelet transform, fractal dimension thresholding

## Abstract

Phonocardiography is a non-invasive technique for the detection of fetal heart sounds (fHSs). In this study, analysis of fetal phonocardiograph (fPCG) signals, in order to achieve fetal heartbeat segmentation, is proposed. The proposed approach (namely WT–FD) is a wavelet transform (WT)-based method that combines fractal dimension (FD) analysis in the WT domain for the extraction of fHSs from the underlying noise. Its adoption in this field stems from its successful use in the fields of lung and bowel sounds de-noising analysis. The efficiency of the WT–FD method in fHS extraction has been evaluated with 19 simulated fHS signals, created for the present study, with additive noise up to (3 dB), along with the simulated fPCGs database available at PhysioBank. Results have shown promising performance in the identification of the correct location and morphology of the fHSs, reaching an overall accuracy of 89% justifying the efficacy of the method. The WT–FD approach effectively extracts the fHS signals from the noisy background, paving the way for testing it in real fHSs and clearly contributing to better evaluation of the fetal heart functionality.

## Introduction

Fetal heart rate (fHR) observation is important for proper fetal well-being assessment during the period of pregnancy. Electronic fetal monitoring (EFM) is a significant tool for the obstetrician, in order to perform various tests at different stages of gestation to estimate the fetal health. The typical examination until the 28th week of pregnancy is composed of continuous measurements of the fetus growth, while at the stage of 29–40 weeks, the monitoring of fetal movement, fetal respiration, fHR, and others (Adithya et al., 2017) are included. In current practice, the examination of the fetus is performed by means of ultrasonic-based equipment such as Doppler ultrasound and cardiotocogram (Nassit and Berbia, 2015).

Although Doppler ultrasound and cardiotocogram are the typical fetal observation devices, these techniques present some limitations, mainly because of the cost of the monitoring devices and the complexity of their use, demanding an expert during data acquisition. Moreover, it has not been established that the frequent and long-term exposure to ultrasound energy has no effect on either the fetus or the mother (Salvesen, 2002).

Existing standards of fetal monitoring estimate the fetus and the mother physiology with repetitive examinations. However, complications may occur during pregnancy, i.e., fetal deaths, preterm delivery, hypoxia, and other, which have no specific prevision. Even though the literature is not robust about the risks and their relation to the EFM, long-term fHR monitoring has proven to be an effective approach for better accuracy in the clinical examination of the fetus (Martin, 1998).

A passive alternative for long-term monitoring of the fetus is the fetal heart auscultation. It is a non-invasive method that records the vibroacoustic signals from the abdominal surface. The acoustic signal produced by the fetal heart sound (fHS) can be visually depicted in the fetal phonocardiograph (fPCG). We can separate the fetal heartbeat into two sub-beats, the systolic beat S1 and the diastolic beat S2, which follows S1. The S1 and the S2 sub-beats are generated by the vibratory components of the fetal heart valves closure. The S2 sub-beats present smoother morphology than the S1, making harder the detection of their location. A heart cycle consists of the S1 and S2 sub-beats.

The research of fPCG signals aims to segment the S1 and S2 sub-beats, in order to study the wavelet morphology of the fHSs and the fHR variability. Long-term monitoring of the fHSs reveals information about the fetus growth and functionality. Although there is not enough knowledge about fHS morphology, in order to indicate any pathological conditions, the study of the fPCG signals have shown promising results to the extension of the EFM and the physical examination of the fetus (Adithya et al., 2017).

Auscultation is a low-cost and non-invasive method as it captures the acoustic signal of the fHSs. Moreover, the phonocardiogram device is a flexible method that does not need an expert to record the signals. The mother can take long-term recordings during the day or night and afterward, the doctor can examine the signals and have a more complete overview of the fetus functionality.

Nevertheless, fetal auscultation has many challenges. Because of the place of the fetus in the maternal abdominal, the fPCG signals are loaded with noise from various sources such as maternal heart sounds, digestive sounds, maternal and fetus respiration movements, external noise, and others (Várady et al., 2003; Cesarelli et al., 2012). In the noisy fPCG signals, the fetal heartbeats are often masked by other components, consequently it is difficult to detect without applying robust signal processing methods.

Throughout the years, various signal processing approaches for de-noising the fPCG signal have been examined and proposed (Unser and Aldroubi, 1996; Messer et al., 2001; Várady et al., 2003; Xiu-Min and Gui-Tao, 2009; Chourasia and Mittra, 2010; Chourasia et al., 2011, 2014). Among them, Khadra et al. (1991) were the first to suggest the wavelet transform (WT) as a useful tool for the analysis of heart sounds. Following, many researchers concentrated on the study of wavelet-based techniques for these signals. Vaisman et al. (2012) proposed the WT as a de-noising tool for the determination of the fHR. At the same time, Kovács et al. (2011) used autocorrelation technique, WT, and matching pursuit for the evaluation of fHS. Recently, Chourasia and Tiwari (2013) designed a new wavelet basis function for de-noising the fPCG signals.

The present study was motivated from a previously proposed method of Hadjileontiadis for the separation of lung and bowel sounds from the background noise (Hadjileontiadis, 2005). The latter technique uses a scheme of WT for de-noising the signals and also fractal dimension (FD) analysis for the detection of lung and bowel sounds. The so-called WT–FD filter introduces an alternative way to the enhancement of bioacoustic signals, applicable to any separation problem involving non-stationary transient signals mixed with uncorrelated stationary background noise (Hadjileontiadis, 2005).

In this study, the WT–FD method is suggested for the case of fPCG signals, to effectively locate and extract the fetal heartbeat from the underlying noise. Due to the highly noisy environment and the low acoustic energy of the fetal heartbeat, WT is an efficient method that decomposes the signal into multiple levels for the subtraction of the unwanted stationary noise. Moreover, the method is flexible since it uses short windows at high frequencies and long windows at low frequencies making the wavelet function more similar to the waveforms of the signal. Furthermore, FD analysis is frequently used in biomedical signal processing. There are studies of FD performance at electroencephalograms for the detection of the onset of epileptic seizures and also at electrocardiogram signals for the classification of arrhythmia with satisfying results (Mishra and Raghav, 2010; Polychronaki et al., 2010).

The rest of the paper is formed as follows. Section “Mathematical Background” describes the mathematical background of WT and FD definitions, while Section “The WT–FD Method” presents the proposed method. Section “Implementation and Evaluation Issues” describes the databases that the method was tested and the general indices that used for its evaluation. Finally, Section “Results” confers some experimental results, which evaluate the efficiency of WT-FD algorithm in fPCG signals, and Section “Concluding Remarks” concludes the paper with suggestions for future work.

## Mathematical Background

### Wavelet Transform

Wavelets are families of functions ψ*_a_*_,_*_b_*(*t*) generated from a single-base wavelet ψ(*t*) called the “mother wavelet,” by dilations and translations (Hadjileontiadis and Panas, 1998; Olkkonen, 2011), i.e.,

(1) $$\psi_{a,b}\left(t\right)=\frac{1}{\sqrt{a}}\psi \;\left(\frac{t-b}{a}\right),\;a>0,\;b\in R,$$

where *a* is the dilation (scale) parameter and *b* is the translation parameter.

In the past few decades, wavelet analysis has been proved to be an important tool in biomedical engineering. The use of WT in fPCG signals is driven by the nature of the signals itself. Explosive peaks in the time domain produce large coefficients over the wavelet scales, while the noisy background dies out swiftly with increasing scale. In WT, the signal is decomposed into coarse and detail information using a pair of finite impulse response filters (and their adjoins), which are low-pass and high-pass, respectively (Hadjileontiadis, 2005). The process can be described as a tree, which at each step decomposes the low-pass filter into further lower and higher frequency coefficients. Thus, the original signal is decomposed into coefficients of lower resolution, and the high frequency coefficients are not analyzed any further. This scheme is a wavelet-based multiresolution decomposition, and it is known as Mallat algorithm (Mallat and Peyré, 2009). The procedure that uses the coarse and the detail coefficients and yields back to the original signal is multiresolution reconstruction.

In the proposed de-noising method, the decomposition– reconstruction scheme was based on the orthonormal bases and the quadrature mirror filters introduced by Daubechies (1988). This wavelet family was chosen because of the morphology of the mother wavelet, comparatively the waveforms of the fPCG and the testing of other wavelet families.

### Fractal Dimension

Fractals are mathematical sets, which describe many natural phenomena with geometrical complexity (Mandelbrot, 1982; Esteller et al., 2001). The term “fractal dimension” can more generally refer to any of the dimensions commonly used for fractals characterization (e.g., capacity dimension, correlation dimension, information dimension, Lyapunov dimension, and Minkowski–Bouligand dimension) (Hadjileontiadis, 2005). More accurately, the FD is a priceless tool that reflects the signal complexity in the time domain. Here, FD was adopted as a means to detect the most important WT coefficients that correspond to the fetal heartbeat in the WT domain, resulting, simultaneously, in significant computational savings.

The FD technique is performed using a sliding window of *W* = int(0.05⋅*F_s_*) samples length, where int(⋅) indicates the integer part of the argument, the constant is empirically set at 0.05 justifying the efficient performance of the algorithm, and *F_s_* denotes the sampling frequency of the signal. It is noticed that when the *W* window is small, too many false FD peaks are generated and when it is big, the estimated FD is smoothed so the algorithm chooses the false peaks.

Let the processing signal be an *N*-sample vector. Then, the *W* -sample window is one-sample shifted along the *N*-sample input vector in order to obtain point-to-point values of the estimated FD. Every estimated FD obtained with the sliding window is assigned to its midpoint. In this way, the length of the final sequence of the FD(*i*) is lower than *N*. This length is extended to comply with the *N*-sample length of the original input vector, assigning the FD(1) and FD(*N* − *W*  + 1) estimated values to the first and last half of the *W*  − 1 missing values, respectively. In this study, we used the Katz’s definition of FD as it is proposed by Hadjileontiadis and Rekanos (2003) for the detection of explosive lung and bowel sounds.

According to Katz (1988), the FD of a curve defined by a sequence of *N* points is estimated by

(2) $$\text{FD}=\frac{\text{lo}{\text{g}}_{\text{10}}\left(n\right)}{\text{lo}{\text{g}}_{\text{10}}\left(\frac{d}{L_{\text{c}}}\right)+\text{lo}{\text{g}}_{\text{10}}\left(n\right)},$$

where *L*_c_ is the total length of the curve, realized as the sum of distances between successive points, i.e.,

(3) $$L_{\text{c}}=\sum_{i=1}^{N-1}\text{dist}\left(i\text{,}i+\text{1}\right),$$

where dist(*i*,*j*) is the distance between the *i* and *j* points of the curve; *d* is the diameter estimated as

(4) d=max[dist(i,j)],i≠j,i,j∈[1,N],

for curves that do not cross themselves; usually, the *d* diameter is estimated as the distance between the first point of the sequence and the point of the sequence that provides the farthest distance, i.e.,

(5) d=max[dist(1,i)],i,j∈[2,N],

and *n*_s_ is the number of steps in the curve, defined as

(6) $$n_{\text{s}}=\frac{L_{\text{c}}}{\alpha },$$

where α denotes the average step, i.e., the average distance between successive points.

## The WT–FD Method

### WT–FD Iterative Procedure

The WT–FD method is an iterative procedure performed in order to achieve the best separation of fetal heartbeat from the superimposed noise. The amplitude normalized *N*-sample input vector *X*[*n*] (*n* = 1, …, *N*), is subjected to the WT–FD technique and is separated into two parts, i.e., $X_{S}^{k}\left[n\right]$ and $X_{U}^{k}\left[n\right]$, the non-stationary desired signal and the stationary background noise, respectively. After that, the process continues iteratively with the vector $X_{U}^{k}\left[n\right]$ serving as a new *X*[*n*] input signal to the next iteration, and the resulted vectors across all *L* iterations, i.e., $X{\left[n\right]}^{1:L}$, are used for the final reconstruction (Hadjileontiadis, 2005). The iterative procedure stops when the following stopping criterion is satisfied:

(7) $$\text{STC}=\left|E\left\{X_{U}^{k-1}{\left[n\right]}^{2}\right\}-E\left\{X_{U}^{k}{\left[n\right]}^{2}\right\}\right|<\varepsilon k\text{-th iteration,}\;n=1\ldots N$$

where *E*{⋅} denotes the expected value. The parameter ε is a small positive number $\left(0<\varepsilon \ll 1.0\right)$ that corresponds to the desired accuracy in procedure. The initial value of $X_{U}^{0}\left[n\right]$ is considered to be equal to 0. When the STC criterion is satisfied after *L* iterations, the final reconstruction of the signal is achieved with the $X_{S}^{k}\left[n\right]$ vectors as follows:

(8) $$X_{\text{REC}}\left[n\right]=\sum_{k=1}^{L}X_{S}^{k}\left[n\right],\;n=1,\ldots ,N,$$

A schematic representation and further details about the WT–FD filter can be found in Hadjileontiadis (2005).

### WT Coefficient Estimation and Selection

**Figure 1:**
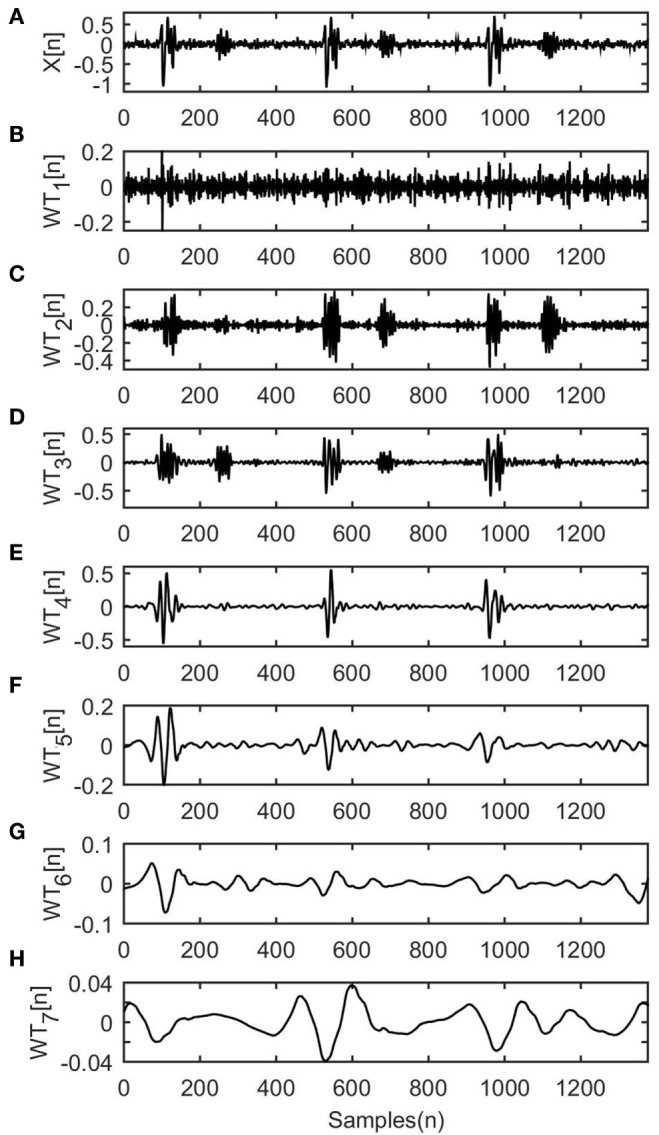
Wavelet transform (WT) decomposition on a simulated fetal phonocardiograph signal. **(A)** The original signal *X*[*n*]. **(B–H)** The seven decomposed levels of the input signal.

In this study, the Daubechies 4 wavelet family (Daubechies, 1988) has been chosen for de-noising the signal. As described in Section “Wavelet Transform,” WT decomposes the input fPCG signal *X*[*n*] (*n* = 1, …, *N*) into *R* detail coefficients WT*_m_*[*n*] (*m* = 1, …, *R*). The number *R* of the adjustment resolution scales is estimated by log_2_ *N*. An example of an fPCG signal is presented in Figures 1A–H, where the original signal is decomposed into seven levels. It is clearly depicted that the first WT level contains only noise and the last three do not contain any important components of the signal. Hence, from the *R* estimated coefficients the algorithm selects those including important information and leaves out those including background noise, as described next.

First, from the *R* estimated WT resolution levels, the first *D* ones are discarded according to the following criterion:

(9) $$D=\text{min}\left\{\lambda :\eta_{1}^{\prime }-\eta_{\lambda +1}^{\prime }\leq 0.4\right\},$$

and from the *J* = (*R* − *D*) coefficients, the first *M* ones are selected according to the following criterion (Hadjileontiadis, 2007):

(10) $$M=\text{min}\left\{\lambda :\left|\eta_{\lambda }^{\prime }\right|>{p\;}^{\wedge }\left|{\eta^{\prime }}_{\lambda +1}\right|\leq {p\;}^{\wedge }\;\eta_{\lambda }^{\prime \prime }>0\right\},$$

with

(11) $$\eta_{\lambda }=1-\frac{\sum_{i=1}^{\lambda }E\left\{\text{W}{\text{T}}_{i}{\left(n\right)}^{2}\right\}}{\sum_{i=1}^{J}E\left\{\text{W}{\text{T}}_{i}{\left(n\right)}^{2}\right\}},\;\lambda =1,2,\ldots ,J\;n=1,2,\ldots ,N,$$

where $\eta \prime_{\lambda }$ and $\eta {\prime \prime }_{\lambda }$ denote, respectively, the first and second derivatives of η_λ_ with respect to λ, *p* is a small number close to 0 that serves as a threshold, which accounts for the fluctuation of the first derivative around 0, and *E*{⋅} denotes the expected value; here, *p* was empirically set equal to 0.01.

### FD-Based S1 and S2 Selection

**Figure 2:**
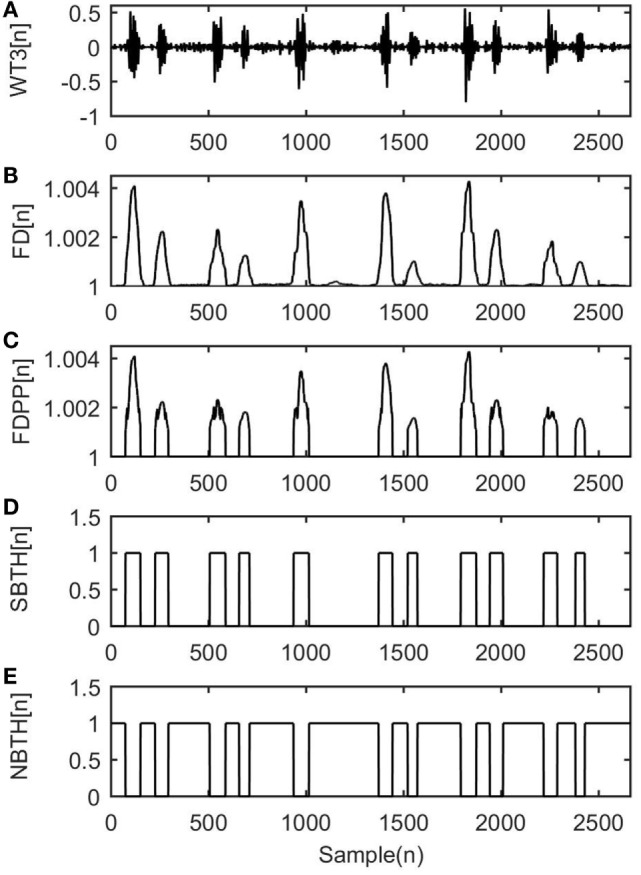
A working example of the production procedure of the binary thresholds ${\text{SBTH}}_{3}^{1}$ and ${\text{NBTH}}_{3}^{1}$ derived from the application of the wavelet transform (WT)–fractal dimension (FD) filter to a case of fetal phonocardiograph recording. These results refer to scale *j* = 3 and iteration *k* = 1 during the application of the WT–FD filter to the input signal. **(A)** WT_3_, the third WT coefficient, **(B)** ${\text{FD}}_{3}^{1}$, the estimated FD using Katz’s definition by Eq. 2, **(C)** ${\text{FDPP}}_{3}^{1}$, the output of the FD-peak peeling algorithm, **(D)** ${\text{SBTH}}_{3}^{1}$, the signal binary threshold, and **(E)** ${\text{NBTH}}_{3}^{1}$, the noise binary threshold.

The fHS segmentation is performing using the FD method across the selected WT*_j_*[*n*] (*j* = 1, …, *M*) WT level. Specifically, the windowing Katz definition of FD as it is described in Section “Fractal Dimension” is performed at every selected coefficient. Then, the estimated ${\text{FD}}_{j}^{i}\left[n\right]\;\left(i\text{-th iteration},\;j\text{-th selected coefficient}\right)$ are fed to the FD-peak peeling algorithm (FD-PPA), as it is proposed by Hadjileontiadis (2005), in order to automatically detect the ${\text{FD}}_{j}^{i}$ peaks. Through a self-adjusted iterative procedure, the FD-PPA iteratively “peels” the estimated FD signal, gradually gathering those parts that construct its peaks, resulting in the ${\text{FDPP}}_{j}^{i}\left[n\right]$ sequence as it is shown in Figures 2A–C. Hence, the algorithm aims to search for the lower peaks, such as the S2 fetus heartbeats, which correspond to the low amplitude coefficients.

In the present study, each WT*_j_*[*n*] (*j* = 1, …, *M*) coefficient is separated in smaller epochs for better FD assessment. Therefore, the FD estimation is more accurate considering the lower peaks. The normal duration of a fetal heart cycle is 430 ms and, consequently, a mean value for each epoch is at 430 ms in order to contain at least one S1 and one S2 heartbeat. Every epoch is fed in the FD-PPA iteration procedure and then reunited in the WT*_j_*[*n*] (*j* = 1, …, *M*) coefficient estimation.

The FD-PPA iteration procedure starts with a threshold operation based on the SD of the vector ${\text{FD}}_{j}^{i}\left[n\right]$ as follows:

(12) $${\text{pFD}}_{j}^{i}=\left\{\begin{array}{cc} {\text{FD}}_{j}^{i}, & {\text{FD}}_{j}^{i}>\mu^{i}+\sigma^{i}\\ \text{1.0,} & \text{elsewhere} \end{array}\right.\text{,}i=1,\ldots ,L_{1};\;j=1,\ldots ,M,$$

where $\mu^{i}=\text{mean}\left({\text{FD}}_{j}^{i}\right)$ is the mean value of the ${\text{FD}}_{j}^{i}$ vector, $\sigma^{i}=\text{std}\left({\text{FD}}_{j}^{i}\right)$ is the SD of the ${\text{FD}}_{j}^{i}$ vector, and *L*_1_ is the number of the self-adjusted iterations. Thus, the vector $z^{i}={\text{FD}}_{j}^{i}-{\text{pFD}}_{j}^{i}+\mu^{i}$ is created, and 1.0 is the minimum value of the estimated FD sequence. The iterative procedure stops when the following stopping criterion is satisfied:

(13) $$\text{S}{\text{C}}^{i}=\left|E\;\left\{{\left(z^{i}\right)}^{2}\right\}-E\;\left\{{\left(z^{i-1}\right)}^{2}\right\}\right|<\text{acc},\;i=1,...,L_{1},$$

where *E*{⋅}, as in the former stopping criterion, denotes the expected value, the parameter acc is a small positive number (0 < acc ≪ 1.0) that corresponds to the desired accuracy in the procedure, and the initial value of *z*^0^ is equal to 0. When the stopping criterion is not satisfied, the vector ${\text{FD}}_{j}^{i}$ is replaced by the vector *z^i^*, and it continues the iterative procedure. When the stopping criterion is satisfied, the FD-PPA generates the ${\text{FDPP}}_{j}^{k}$ sequence of the *j*-th WT coefficient as follows:

(14) $${\text{FDPP}}_{j}^{k}=\sum_{i=1}^{L_{1}}\text{pF}{\text{D}}^{i}-\left(L_{1}-1\right),\;k=1,...,L;\;j=1,...,M,$$

where *L* is the iteration number of the procedure that is described in Section “WT–FD Iterative Procedure.” After the FD-PPA implementation, the small peaks that do not correspond to any sound and their duration is less than int(0.015*F_s_*), and their normalized amplitude less than 0.25 are removed. Again, int(⋅) indicates the integer part of the argument, the constant is empirically set at 0.015, and *F_s_* denotes the sampling frequency of the signal. Subsequently, the ${\text{FDPP}}_{j}^{k}$ sequence is generated and thereafter two binary thresholds are constructed, as shown in Figures 2D,E. The first binary threshold, i.e., ${\text{SBTH}}_{j}^{k}$ is used for segmenting the WT coefficients that are related to the desired signal, while the second one, i.e., ${\text{NBTH}}_{j}^{k}$ is used for segmenting the WT coefficients that are related to the background noise. These two binary thresholds are defined as follows:

(15) $${\text{SBTH}}_{j}^{k}=\left\{\begin{array}{c} 1,{\text{FDPP}}_{j}^{k}\neq 1\\ 0,{\text{FDPP}}_{j}^{k}=1 \end{array}\right.\;\text{,}$$

(16) $${\text{NBTH}}_{j}^{k}=\left[\text{1}-{\text{SBTH}}_{j}^{k}\right]\text{,}\;k=1,...\text{,}L\text{;}\;j=1,\ldots \text{,}M\text{,}$$

The multiplication of the ${\text{SBTH}}_{j}^{k}$ with the WT coefficient gives a set of de-noised signals that create the $X_{S}^{k}\left[n\right]$ vectors as defined in Section “WT–FD Iterative Procedure,” while the multiplication of the ${\text{NBTH}}_{j}^{k}$ with the WT coefficients gives the set of the $X_{U}^{k}\left[n\right]$. Figures 2A–E gives an example where a working scheme of the proposed method is presented on the third WT level of an input signal. It shows that the FD method successfully detects the location of the sounds by using the binary sequences, and it separates the non-stationary bioacoustics signal from the stationary background noise.

In this study, the final goal is to segment the fHS and separate the S1 from the S2 beats. The decision between S1 and S2 is based on the fact that in a cardiac cycle the diastolic duration is longer than the systolic one (Papadaniil and Hadjileontiadis, 2014). For that reason, the following inequality is checked:

(17) S(2i+1)−S(2i)<S(2i+3)−S(2i+2),

where *S*(*l*) is a vector that is created by the binary threshold ${\text{SBTH}}_{j}^{k}$, and it contains the locations of the start and the end of every fetal heartbeat. Moreover, *i* = 1, …, (*N*_1_/2) − 2, where *N*_1_ is the length of the *S*(*l*) vector. If Eq. 17 is true, the interval [*S*(2*i* + 1):*S*(2*i* + 2)] corresponds to S2, otherwise, it corresponds to S1. The first and the last heartbeat of the signal are not determined from this inequality. Hence, they need to be separately defending. For *i* = 1, if Eq. 17 is true, then the second sound [*S*(4):*S*(5)] is S2 and the first sound [*S*(1):*S*(2)] is S1. Respectively, for *i* = (*N*_1_/2) − 2, if Eq. 17 is true, the last sound [*S*(*N*_1_ − 1):*S*(*N*_1_)] is defined as S1.

A criterion of each estimated fetal heartbeat amplitude and the distance between fetal heart cycles is also considered for better decision between S1 and S2 beat. In the literature, the mean amplitude of a fetal S1 beat is equal to 0.7 (Cesarelli et al., 2012), and the distance between fetal heart cycles, i.e., between S2 and the following S1, depends on the fHR. The smaller distance between fetal heart cycles is in case of tachycardia and is about 140 ms. Thus, for the decision between S1 and S2 beat, the S1 estimated beat must surpass the 0.5 normalized amplitude and the S2, S1 inter-distance must be outdistance within 130 ms.

## Implementation and Evaluation Issues

The analysis of this study was applied on a personal computer using Matlab R2015a and tested on simulated databases. Every input signal was tested for 10 s considering *F_s_* = 1,000 Hz, i.e., 10,000 samples.

For the purposes of this research and the algorithmic development of the WT–FD method, a database with fPCG signals was created. Each signal contains simulated S1 and S2 auscultation sounds created by Hadjileontiadis using the model of Chen et al. (1997) and Xu et al. (2001) and adjusted to the duration of fetal heartbeat. The inter-distance between S1 and S2 heart sounds is given by the expression SSID = 210 − 0.5 ⋅fHR according to Kovacs et al. (2000). Moreover, in order to represent the noise presence, additive white Gaussian noise was used, resulting in signal-to-noise-ratio (SNR) within the range of SNR = [8, 3] dB. The SNR values were computed according to the following steps; measure the power of the signal (*P_s_*), convert the given SNR in decibels (SNR_dB_) to linear scale according to $\text{SN}{\text{R}}_{\text{linear}}=10^{\frac{\text{SN}{\text{R}}_{\text{dB}}}{10}}$, and finally create the noise vector from Gaussian distribution of specific noise variance according to $\text{noise}=\sqrt{\frac{P_{S}}{\text{SN}{\text{R}}_{\text{linear}}}}\;\cdot \text{random}$, where random is a vector of normally distributed random numbers with the signal length.

The database consists of signals with different heart conditions corresponding to cases such as tachycardia, bradycardia, and arrhythmia. Specifically, after the 20th week of gestation, the fHR is stabilized between the 110 and the 160 bpm. Thus, for the normal heartbeat signals, the fHR was set at 140 bpm, for the bradycardia signals at 110 bpm, for the tachycardia signals at 180 bpm, and for cases of arrhythmia a range of 80–200 bpm was considered. Hence, many signals with different conditions and different values of additive white Gaussian noise were created and used for testing the present study.

Furthermore, for better assessment of the WT–FD technique, the method was tested on the simulated fPCGs database available *via* PhysioBank.^1^ PhysioBank is a large archive of digital recordings of physiological signals and related data for use by the biomedical research community (Goldberger et al., 2000). The simulated fPCG database was created by Cesarelli et al. (2012) and Ruffo et al. (2010). This data set is a series of synthetic fPCG signals related to different fetal states and recording conditions. Simulated fPCG were generated as a sequence of frames, each of which includes simulated S1 and S2 signals, corrupted by noise. These signals are qualified by a range of SNR values that were computed in decibels according to the following formula:

(18) $$\text{SNR}=\text{10lo}{\text{g}}_{\text{10}}\left(\frac{P_{s}}{P_{n}}\right),$$

where *P_s_* and *P_n_* are the power of fHS and the power of the noise, respectively. The noise source was simulated by generating maternal and fetal noise, maternal first heart sound, white Gaussian noise, environmental noises, and limited duration impulses considering as sensor noises. The epoch lengths were set equal to 430 and 400 ms for the analysis of the PCG drawn from the two databases, respectively, using in both databases a window length of 50 samples.

### General Evaluation Indices

The effectiveness of the WT–FD technique was tested *via* three general evaluation indices. The first *Q_P_* index calculates the efficiency of the algorithm in the correct detection of the S1 and S2 fetal heartbeat and its performance in the detection of locations that are not related with existing sounds. The *Q_P_* index is defined as follows:

(19) $$Q_{P}=\text{100}\sqrt{\frac{S_{C}}{S_{O}}\frac{S_{C}}{S_{P}}}\text{,}$$

where *S_O_* is the number of sounds that every record contains, *S_P_* is the number of sounds that the proposed algorithm detects, and *S_C_* is the number of the *S_P_* sounds matching the *S_O_* sounds. Since the signals are simulated, the location of the existing sounds is specific, i.e., the *S_O_* number. For the *S_C_* number, the fHS was assumed to have been correctly detected when the estimated peaks lied in the intervals [*S*(2*i* + 1):*S*(2*i* + 2)], i.e., the start and end of each existing heart sound.

Furthermore, the second *D_R_* index indicates the percentage of the sounds that the WT–FD algorithm correctly detects out of the total number of sounds that it detects. The *D_R_* index is defined as follows:

(20) $$D_{R}=\frac{S_{C}}{S_{P}}100.$$

Conclusively, the third *S_F_* index indicates the percentage of the sounds that the algorithm detects correctly out of the real fHS that every record contains. The *S_F_* index is defined as follows:

(21) $$S_{F}=\frac{S_{C}}{S_{O}}100.$$

The above three indices were calculated for the evaluation of the testing WT–FD method, and the results are presented in Section “Results”.

## Results

As mentioned in Section “General Evaluation Indices,” the WT– FD technique was tested on two simulated databases of fPCG signals. The results of this assessment are presented in Tables 1 and 2 where the *Q_P_*, *D_R_*, and *S_F_* indices are tabulated, providing a means for the evaluation of the performance of the WT–FD algorithm for the detection of the fetal S1 and S2 heartbeat and also each fHS separately.

**Table 1:** Performance of the wavelet transform–fractal dimension filter for cases of simulated fetal phonocardiograph signals created by Hadjileontiadis.

Fetal heart rate	Signal-to-noise-ratio	S1, S2			S1			S2		
		*Q_P_*%	*D_R_*%	*S_F_*%	*Q_P_*%	*D_R_*%	*S_F_*%	*Q_P_*%	*D_R_*%	*S_F_*%
140	8	100	100	100	100	100	100	100	100	100
	5	100	100	100	100	100	100	100	100	100
	3	100	100	100	100	100	100	100	100	100
110	8	97.3	100	94.7	100	100	100	94.6	100	89.5
	5	97.3	100	94.7	100	100	100	94.6	100	89.5
	3	98.7	100	97.3	100	100	100	97.3	100	94.7
180	8	97.5	100	95	98.3	100	96.7	96.6	100	93.3
	5	97.5	100	95	98.3	100	96.7	96.6	100	93.3
	3	95.6	98.2	93.3	96.7	96.7	96.7	94.9	100	90
Arrhythmia	8	92.8	100	86.4	97.7	100	95.5	87.9	100	77.3
	5	94.1	100	88.6	97.7	100	95.5	90.5	100	81.8
	3	92	100	84.1	97.7	100	95.5	85.3	100	72.7

**Table 2:** Performance of the wavelet transform–fractal dimension filter for signals of PhysioBank.

Signal-to-noise-ratio	S1, S2			S1			S2		
10log_10_$\left(\frac{{\text{P}}_{\text{s}}}{{\text{P}}_{\text{n}}}\right)$	*Q_P_*%	*D_R_*%	*S_F_*%	*Q_P_*%	*D_R_*%	*S_F_*%	*Q_P_*%	*D_R_*%	*S_F_*%
−6.6	94.9	100	90	100	100	100	89.4	100	80
−11.3	96.6	100	93.3	100	100	100	93	100	86.7
−15.7	87.8	92.6	83.3	93	100	86.7	82.8	85.7	80
−17.2	88.1	89.7	86.7	93	100	86.7	83.9	81.2	86.7
−22.1	68.9	67.7	70	89.4	100	80	53.3	47.4	60
−24.4	66	93.3	46.7	85.6	100	73.3	38.7	75	20
−26.3	62	82.4	46.7	77.5	100	60	45.6	62.5	33.3

In particular, Table 1 presents the cases of 12 simulated fPCG signals created for the present study and consists 4 different fHRs and 3 different SNR values (white Gaussian noise). From Table 1 it is clear that the WT–FD method is efficient for multiple conditions. The *Q_P_* index indicates that in all cases of fHR the WT–FD correctly predicts almost all the observed sounds in different SNR values up to 3 dB. Specifically, in cases of normal fHR (140 bpm), the algorithm has mean performance 100%. In cases of tachycardia (180 bpm), bradycardia (110 bpm), and arrhythmia, the efficiency of the method is slightly lower although it is sufficiently effective in the detection of the S1 beat locations.

Moreover, Table 2 presents the cases of seven simulated fPCG signals from PhysioBank with different SNR values. Results for the cases of normal fHR with a range of SNR noise lying in [−26.3, −6.6 dB] demonstrate that the WT–FD algorithm segments and detects almost all the observed heart sounds and has a mean accuracy 81%. However, it is clear that the lower the SNR value, the harder it is for the WT–FD to segment and select the correct S2 fetal heartbeat. Very low SNR (less than −22.1 dB) makes the S2 sound difficult to distinguish from the noise. The *D_R_* index declares that, despite the fact that the algorithm misses a few heartbeats, it does not detect false locations. Most of the detected sounds are assigned to real fetal heartbeat locations. Furthermore, it is notable that all the detected S1 beat locations refer to real sounds.

**Figure 3:**
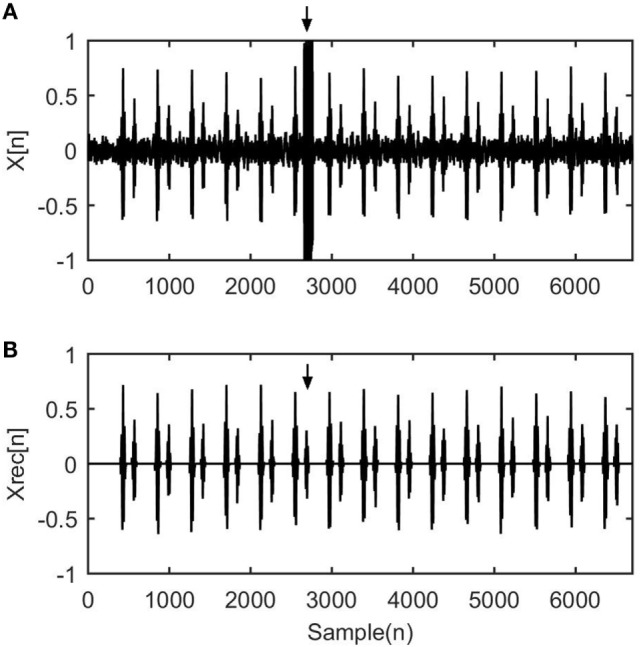
Experimental result from the application of the wavelet transform–fractal dimension scheme to simulated fetal phonocardiograph signal. **(A)** *X*[*n*] represents a section of 5,000 samples of a normal heart rate case with unexpected robust noise. **(B)** *X*_REC_[*n*] corresponds to the normalized treated signal without the overlap of noise. The arrows indicate the location of the S2 sound that the algorithm efficiently reveals.

Figure 3 shows the efficiency of the WT–FD technique to recognize the fHS in signals with unexpected noise presence. Figure 3A corresponds to the *X*[*n*] unprocessed signal, and Figure 3B corresponds to the *X*_REC_[*n*] segmented reconstructed signal. In *X*[*n*] signal it is obvious that there is a noisy segment, which is marked with an arrow, that masked the S2 heart sound. In the *X*_REC_[*n*] signal it is clear that the WT–FD successfully extracts the sound.

The proposed WT–FD approach was also tested in real fPCG signals from a small pilot study, involving recordings from three pregnant women. The fPCG signals were recorded using vibration sensors (cost $1 each) embedded in high definition 3D-printed plastic harnesses. Each harness holds a ceramic piezo vibration sensor (35 mm diameter) on the maternal abdomen with rubber-made cushion to minimize the shear noise. The 3D-printed harness is designed with precise parameters that rigidly mount the piezo sensor. Each sensor picks fPCG signals through a coaxial cable having very high insulating resistance. Power lab data acquisition system by AD instrument^2^ was used to record the abdominal phonograms at a sampling frequency of *f_s_* = 1,000 Hz.

**Figure 4:**
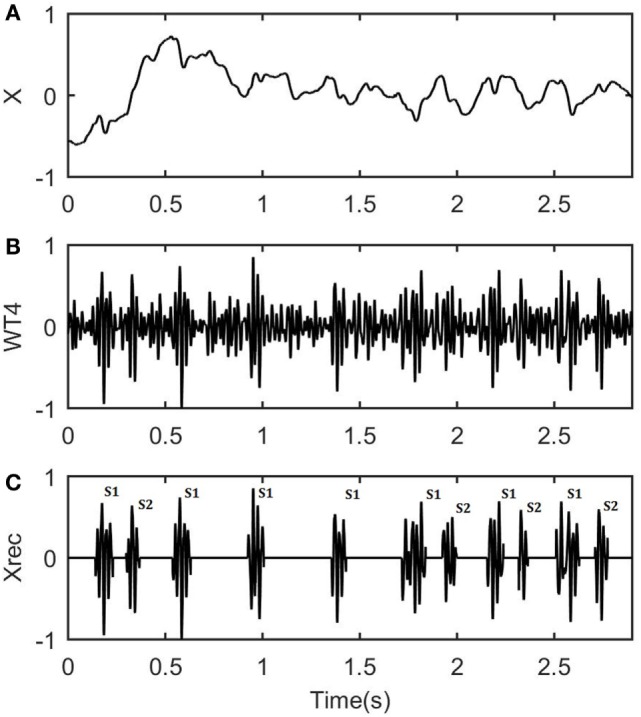
Analysis results when wavelet transform (WT)–fractal dimension (FD) filter is applied to a part of real data. **(A)** A time section of 3 s of the real fetal phonocardiograph recording, with maternal heart rate of 96 bpm and fetal heart rate of 145 bpm. **(B)** The fourth level of the estimated WT coefficients selected for the detection of the fetal heart sounds (fHSs). **(C)** The result of the de-noised fHS signal after the final WT–FD analysis with S1 and S2 denoting the first and second fHS, respectively.

A characteristic example of one channel fPCG recording (time section of 3 s) with maternal heart rate of 96 bpm and fHR of 145 bpm is shown in Figure 4A. From the latter, it is clear that the fPCG signal is modulated by noise from various sources, and the most intense interferences are the mother’s respiratory and heart sounds. Figure 4B shows the fourth level of the estimated WT coefficients from the eight level WT decomposition. The WT–FD method selects these WT coefficients that include information regarding the signal of interest, i.e., fHSs, based on the criterion (Eq. 9). For the real fPCG data processing, the constant of the criterion was set at 0.001, leaving out the first three decomposition levels and including only those with embedded fHSs. Finally, Figure 4C depicts the estimated fHS signal, i.e., the detected S1 and S2 fHSs, marked with (S1) and (S2), respectively, as the final output from the proposed WT–FD method. Note that, in some cases [Figure 4C around (0.5–1.5 s)], three S2 fHSs were missed by the WT–FD filter due to their lower intensity, compared to the neighboring S1 ones and the local background noise. Nevertheless, when comparing the original recording of Figure 4A with the outputted fHS signal from the WT–FD approach in Figure 4C, a clear contribution to the enhancement of the fHS signal from its original recording is evident.

It should be noted that the fHSs are not perfectly periodic due to the heart rate variability. It can be seen that, despite the noisy signal, the WT–FD method successfully identifies the fHSs and their time location and duration, giving the physicians the means to estimate the fHSs and the fHR. The wavelet morphology of the sounds could vary with different pathophysiological conditions. This is of great importance when the fHSs are continuously recorded for long-term analysis.

## Concluding Remarks

The fPCG signals are of low amplitude and loaded with heavy noise. The sources of the noise, i.e., maternal sounds, fetal movement, sound produced by the transducer, and other, are overlapping the main fHS. The literature in the area of fetal auscultation is not strict about the intensity of background noise and the intensities of S1 and S2 heartbeat, because of the different auscultation devices but also due to the different gestation age. Nevertheless, it is possible to argue that the amplitude of the stationary background noise did not fully overlap the fHS, and that the SNR values that have been tested in the present study were sufficient samples of heavily loaded signals. However, as it was shown by the testing results, the WT–FD scheme is quite satisfactory in the analysis of the fHS. This first approach of the research in fPCG signals reveals sufficient information, which indicates that this technique can be a promising fHS segmentation tool. Furthermore, there are perspectives for low-cost and continuous recordings in homecare setups and diagnosis of conditions related, for example, to fetus maturation or specific abnormalities.

Future work will focus upon the extension of WT–FD to real recorded signals for a better review of fetal functionality and the fetal heart cycle. Moreover, multichannel recordings could be considered, taking into account the spatial orientation of the fetus and the proximity to the mother’s heart sound noise. As phonocardiography has been an important field in the research area related to the fetus for some time, efficient characterization of fetal heartbeat could contribute to the automated determination of fetus parameters. In this vein, the determination of multiple fetus health data may reveal new aspects, which could improve the safety of pregnancies.

## Author Contributions

Producing the code used in the paper: EK and LH. Analyzing the signals and drawing conclusions as well as discussing the structure of the paper and writing up the final version: EK, LH, IC, and AK.

## Conflict of Interest Statement

The authors declare that the research was conducted in the absence of any commercial or financial relationships that could be construed as a potential conflict of interest. The reviewer, WT, and handling editor declared their shared affiliation, and the handling editor states that the process nevertheless met the standards of a fair and objective review.

## Footnotes

^1^https://physionet.org/physiobank/database/simfpcgdb/.

^2^http://www.adinstruments.com.
